# Recent advances in electrospinning nanofiber materials for aqueous zinc ion batteries

**DOI:** 10.1039/d3sc05283d

**Published:** 2023-11-03

**Authors:** Sinian Yang, Shunshun Zhao, Shimou Chen

**Affiliations:** a State Key Laboratory of Chemical Resource Engineering, Beijing Key Laboratory of Electrochemical Process and Technology of Materials, Beijing University of Chemical Technology Beijing 10029 China chensm@buct.edu.cn

## Abstract

Aqueous zinc ion batteries (AZIBs) are regarded as one of the most promising large-scale energy storage systems because of their considerable energy density and intrinsic safety. Nonetheless, the severe dendrite growth of the Zn anode, the serious degradation of the cathode, and the boundedness of separators restrict the application of AZIBs. Fortunately, electrospinning nanofibers demonstrate huge potential and bright prospects in constructing AZIBs with excellent electrochemical performance due to their controllable nanostructure, high conductivity, and large specific surface area (SSA). In this review, we first briefly introduce the principles and processing of the electrospinning technique and the structure design of electrospun fibers in AZIBs. Then, we summarize the recent advances of electrospinning nanofibers in AZIBs, including the cathodes, anodes, and separators, highlighting the nanofibers' working mechanism and the correlations between electrode structure and performance. Finally, based on insightful understanding, the prospects of electrospun fibers for high-performance AZIBs are also presented.

## Abstract 2

This review paper summarizes the application of electrospinning nanofibers in the cathodes, anodes, and separators of aqueous zinc ion batteries.

## Graphical abstract

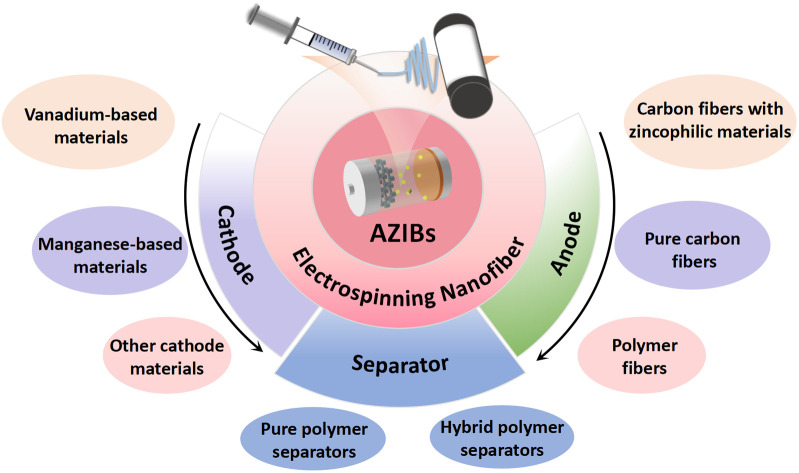

## 1 Introduction

With the rapid consumption of fossil resources and increasing demand for highly efficient utilization of new energy, the search and study of energy storage devices with high earth abundance, good safety, and long cycle life are urgently required.^1–4^ As a promising candidate for large-scale energy storage systems, AZIBs have attracted wide attention due to their rapid reaction kinetics, environmental benignity, and affordability.^5–7^ Generally, AZIBs are composed of a Zn anode, mild or weakly acidic electrolyte, separator, and cathode. Zn metal with a high theoretical capacity (820 mA h g^−1^) and low redox potential (−0.76 V *vs.* the standard hydrogen electrode) is considered an ideal anode for AZIBs.^8,9^ In addition, the cathode plays a crucial role in the performance of AZIBs, as it serves as a host framework to accommodate Zn^2+^.^10,11^ So far, cathode materials for AZIBs include manganese, vanadium, Prussian blue analogs, organic compounds, *etc.*^12^ These cathode materials are related to the operation voltage, cycle stability and rate performance of AZIBs.^13,14^ Herein, the application of suitable cathode materials can improve the performance of AZIBs.

Despite the many advantages of AZIBs, however, many challenges seriously hinder their further application. Firstly, in contrast to lithium/sodium ion batteries, the reaction mechanisms of AZIBs are complicated and immature,^15^ and can be categorized into three main types, including Zn^2+^ insertion/extraction,^16,17^ H^+^/Zn^2+^ co-insertion/extraction,^18^ and chemical conversion reactions.^19^ Among them, the Zn^2+^ insertion/extraction reaction mechanism is the most commonly acknowledged in AZIBs.^20^ Secondly, the non-uniform Zn^2+^ deposition and the decomposition of active H_2_O molecules belonging to the solvation layer of Zn^2+^ will result in uncontrolled growth of Zn dendrites and the formation of by-products on the surface of the Zn anode, ultimately causing battery failure.^21^ Thirdly, due to the Jahn–Teller effect, the active materials of the manganese-based materials will dissolve in weakly acidic aqueous electrolyte, resulting in material collapse and the rapid degradation of capacity.^22,23^ In addition, vanadium-based compounds and organic compounds also face the challenge of dissolution.^24^ Fourthly, as the crucial component of AZIBs, the separator can prevent direct contact between the electrodes and provide the channel for ion migration.^25^ However, traditional separators (such as glass fiber, filter paper, and non-woven fabrics) cannot meet the requirements for AZIBs of excellent mechanical properties, high wettability, high ionic conductivity, and electrical insulation.^26,27^ To alleviate these limitations, some novel material preparation technologies and many functional materials have been adopted and fabricated. Among them, the electrospinning nanofibers have advantages such as large surface area to volume ratio, high aspect ratio, directional transportation, and short ionic transport lengths, which are desirable in energy storage applications.^28^ In the previously reported literature, there is no uniform definition of one-dimensional (1D) nanofibers.^29^ Thereby, in this review, single electrospinning nanofibers are defined as 1D nanofibers. During electrospinning, 1D nanofibers deposited and disorderly arranged on the collector can form the two-dimensional (2D) nano-film. Different from the conventional 2D nano-film, the preparation of three-dimensional (3D) fibrous structures is complicated. In general, the fabrication strategies of 3D structures include increasing the electrospinning, self-assembly, assembly by post-processing of 2D nano-film (such as layer-by-layer electrospinning), and direct assembly by an auxiliary factor (like a 3D template).^30^ These 2D and 3D architecture materials with high flexibility and high surface area-to-mass ratio are assembled by 1D fibers exhibit faster intercalation kinetics in AZIBs. Besides, some unique structures (such as core/shell structures and hierarchical pores), defects, and functional groups can be created and introduced on the electrospinning nanofibers, which is beneficial for AZIBs.^31^

**Fig. 1:**
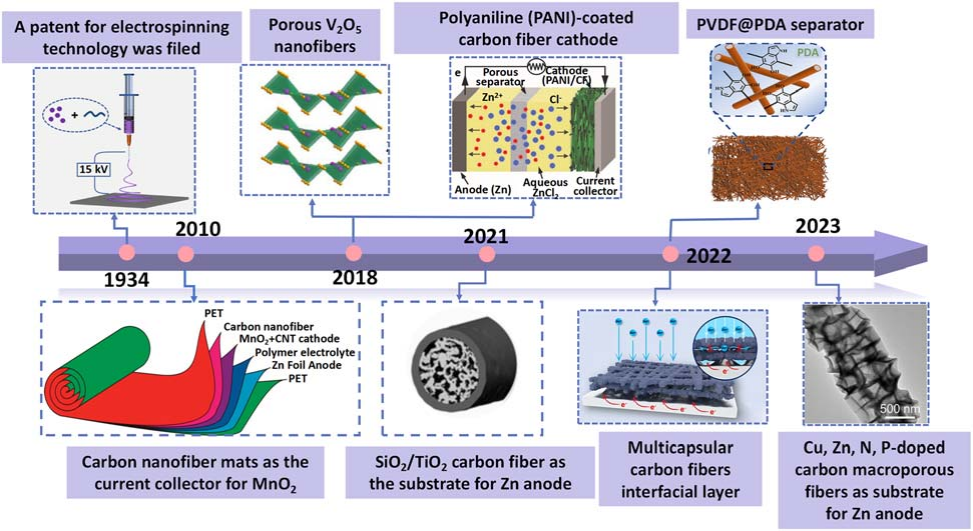
Timeline of the progress of the electrospinning nanofibers in AZIBs. Adapted from ref. 36, copyright 2022, American Chemical Society. Adapted from ref. 37, copyright 2010, American Chemical Society. Adapted from ref. 38, copyright 2019, Elsevier B.V. Adapted from ref. 39, copyright 2018, American Chemical Society. Adapted from ref. 35, copyright 2021, Wiley-VCH. Adapted from ref. 40, copyright 2022, the Author(s). Adapted from ref. 41, copyright 2022, the Author(s). Adapted from ref. 42, copyright 2023, American Chemical Society.

For example, Tang *et al.* fabricated N-doped carbon fibers to improve the electronic conductivity of cathode materials.^32^ Liang *et al.* synthesized zincophilic carbon nanofiber interlayers by an electrospinning method to uniformize the deposition of Zn^2+^.^33^ Meanwhile, Fang *et al.* fabricated a polyacrylonitrile (PAN) nanofiber separator with high porosity and excellent flexibility.^34^ A brief timeline of the representative works of electrospinning nanofibers on AZIBs is summarized in Fig. 1.^35–42^ Although electrospinning nanofibers are widely applied in AZIBs, there is still no specific review focus on electrospinning nanomaterials' application in AZIBs. Thus, it is necessary to summarize the research progress of AZIBs based on the electrospinning nanomaterials.

Herein, in this review, we first introduce the principle and processing of the electrospinning technique. Then, the different structures of electrospinning nanofibers in AZIBs are summarized. Thirdly, we highlight the development of electrospinning materials in AZIBs, such as cathodes,^39,43,44^ anodes,^45–47^ and separators.^34,48^ Finally, we propose the challenges, development prospects, and future research directions of the electrospinning materials in AZIBs.

## 2 Principle and processing of the electrospinning technique

The electrospinning technique is a novel patented technology invented in 1934 that enables the direct and continuous preparation of polymer nanofibers,^49,50^ including not only synthetic polymeric compounds such as poly(vinyl pyrrolidone) (PVP), poly(vinylidene fluoride) (PVDF), and polyacrylonitrile (PAN),^51^ but also natural macromolecules and their derivatives like chitosan and silk protein.^30^ A common electrospinning apparatus usually comprises a high-voltage power supply, a metallic or plastic syringe, and a collector.^31^ A “Taylor cone” at the end of the nozzle will form a jet of electrically conductive polymeric precursor solution (or polymer melt) in a classic electrospinning process when the voltage between the collector and needle exceeds a critical value.^50^ After a short distance of stable motion, these jets will go into an unstable movement stage. Experiencing a series of stretching and solvent evaporation, the polymer solution jets will solidify and finally be deposited on the collector, forming polymer fibers.^52^

The structure and morphology of electrospinning nanofibers are affected by numerous factors such as the properties of polymer solutions, processing parameters, and ambient parameters.^53^ The molecular weight of the polymer is a significant parameter affecting electrospinning nanofibers, which directly affects the properties of the precursor solution, such as viscosity, conductivity, and surface tension.^54^ At the same mass fraction, polymer solutions with higher molecular weight exhibit higher viscosity than those with lower molecular weight. In general, high viscosity usually results in the formation of large diameter nanofibers, while low viscosity solutions facilitate the preparation of small diameter nanofibers.^50^ Voltage and feed rate are other important factors affecting the diameter of the nanofibers. It is well known that the critical voltage is required to form electrospinning nanofibers.^55^ With the increase of voltage, the diameter of nanofibers will decrease at an appropriate concentration of polymer solution.^52^ In contrast, increasing the feed rate will lead to an increase in fiber diameter.^56^ Besides, the diameter of nanofibers is also influenced by environmental parameters (such as humidity and temperature). A moderately high temperature and a low relative humidity will promote the evaporation of solvent and the solidification of jets, which is favorable for decreasing the diameter of nanofibers. These factors are not independent and have a significant influence on each other. Therefore, before preparing the nanofibers with specific morphology and diameter, the interaction between these parameters needs to be considered.

## 3 Electrospinning nanofibers design for zinc ion batteries

Generally speaking, the structure of materials significantly impacts the electrochemical performance of batteries. For instance, constructing a porous structure cathode material can increase the SSA of the material and facilitate the intimate electrolyte penetration and rapid transfer of Zn^2+^.^57^ Furthermore, the hollow structure has the ability to accommodate the volume changes of the electrodes.^58^ Different structures of nanofibers (*e.g.*, core/shell, porous, hollow, and so on) can be fabricated by the electrospinning method. Thus, the design of different structure nanofibers by electrospinning in AZIBs will be discussed and summarized in this section.

### 3.1 Core/shell structure

In the year 2003, nanofibers with core/shell structures were prepared by coaxial electrospinning for the first time.^59^ Since then, the core/shell-structured electrospinning nanofibers have been extensively utilized in energy storage due to their unique features. Compared with normal electrospinning fibers, the advantage of core/shell nanofibers is to allow many non-spinnable polymers to be used as electrospinnable materials,^60^ such as polyaniline and polyvinyl alcohol.^61^ In the process of electrospinning, two kinds of immiscible solutions were added to two syringes, respectively. Then, under a high voltage electrostatic field, the shell solution will converge with the core solution at the nozzle, finally forming the core/shell structured fibers.^62^ In AZIBs, the core/shell nanostructure fibers are usually used as the electrode material due to the large SSA and excellent charge storage. For example, Long *et al.* fabricated Mn_3_O_4_ nanoparticles (Mn_3_O_4_ NPs)/polyacrylonitrile (PAN) composite nanofibers by coaxial electrospinning.^63^ During annealing, the Mn_3_O_4_/PAN fibers were carbonized to Mn_3_O_4_@HCFs nanofibers with core/shell structure.

### 3.2 Porous structure

Porous structure electrospinning nanofibers have the advantages of large SSA, short ion diffusion length, and fast electrolyte access, and have been widely used in AZIBs.^64^ Besides, the abundant porosity can accommodate the volume changes caused by ion insertion/extraction, thus mitigating structural distortion during cycling.^65^ In electrospinning, phase separation and sacrificial template methods are usually used to produce porous structures in nanofibers. The mechanism of the phase separation method can be categorized into vapor-induced phase separation (VIPS), non-solvent-induced phase separation (NIPS), and thermally induced phase separation (TIPS).^66^ Usually, the fabrication of porous nanofibers involves one or more phase separation methods, while suitable polymers and solvents are also required. Sacrificial templates include polymers, metals, metal oxides, and inorganic salts.^67^ For instance, Liu's group used block copolymer poly(methyl methacrylate)-*block*-polyacrylonitrile (PMMA-*b*-PAN) as a raw material to fabricate polymer mats.^68^ In this polymer mat, the incompatibility between the PMMA block and PAN will result in microphase separation, which will further release and generate abundant micro-/mesopores at high temperatures. This porous structure can shorten the ion diffusion path and facilitate the migration of electrolytes in the electrode.

### 3.3 Hollow structure

The principle of coaxial electrospinning to prepare hollow structure nanofibers involves generally soluble or volatile substances (such as oil) as the core layer, and polymer solution as the shell layer, through the coaxial electrospinning process and removal of the core layer to obtain hollow fibers.^69,70^ The construction of hollow structures can significantly increase the number of active sites, improve the high aspect ratio of nanofibers, and enable accommodating massive deposition at a high current density without a distinct volume change. Additionally, it can be prepared by the Kirkendall effect.^71^ For example, Xue *et al.* proposed a hollow TiO_2_ and SiO_2_ carbon fiber. During the carbonization process, hollow porous fibers were formed due to the different decomposition and diffusion rates of different molecular weight PVP.^35^

### 3.4 Bead-like structure

In recent years, the bead-like structure of electrospinning nanofibers has attracted extensive attention on account of its unique geometric shape and chemical performance. Usually, bead-like structure fibers are considered the by-products of the electrospinning process. Their formation can be devoted to the axisymmetric instability of the fluid jet under an external electric field.^72,73^ According to the literature, decreasing the viscosity of the polymer solution (or net charge density of the jets) will facilitate the formation of beads.^74^ However, the lower surface tension of the precursor polymer solution favors the production of bead-like fibers during the process of electrospinning. For instance, the manganese-based metal–organic framework (Mn-MOF) spheres can be wrapped in PAN through the electrospinning technique.^36^ After carbonization in N_2_, the bead-like cathode materials for AZIBs can be achieved by stringing MnO_*x*_ with carbon fiber ropes.

### 3.5 Hierarchical structure

Hierarchically structured fibers consist of multiple nanostructures, which can be fabricated by electrospinning and post-treatment technologies.^50^ Compared to primary structures, the hierarchical structure improves the electrical conductivity of metal oxides and the storage of Zn^2+^.^75^ For instance, Zhang *et al.* produced vanadium nitride embedded nitrogen-doped carbon nanofiber (VN/N-CNFs) composite hierarchical structures by the electrospinning method.^76^ Additionally, nano-whiskers can be observed in the branches of VN/N-CNFs.

## 4 Applications of electrospinning nanofibers in zinc ion batteries

Owing to their versatility and applicability, electrospinning nanofibers have been extensively applied in AZIBs. Firstly, electrospinning nanofibers possess high mechanical flexibility to meet the trend of flexible AZIBs. Secondly, the nanofiber structure can shorten the Zn^2+^ diffusion pathway and reduce reaction impedance in cycling. Thirdly, electrospinning nanofibers with electrical conductivity and stability can be used as a collector to uniformize the deposition of Zn^2+^, achieving a “dendrite-free” metal Zn anode. Last but not least, the nanofiber separator with appropriate thickness, high mechanical strength, and controllable pore size can be fabricated by the electrospinning technique, which can facilitate the transfer of Zn^2+^, improve the wettability between the separator and electrolyte, and resist the piercing of the Zn dendrites. Therefore, this section will summarize the application of electrospinning nanofibers in the cathodes, anodes, and separators of AZIBs.

### 4.1 Cathodes

In particular, as an important component of AZIBs, the cathode material largely determines the electrochemical behaviors of the battery.^77^ Therefore, high-performance cathode materials have been the focus of research in the last decade.^78^ However, cathode materials still face challenges such as poor conductivity, dissolution issues, and volume variation.^23,79^ Electrospinning carbon nanofibers can provide carbonaceous frameworks with high conductivity to improve the conductivity and reaction kinetics of materials.^55,80^ Besides, the active materials can be embedded in carbon nanofibers with a porous structure and large SSA, which greatly prevents the dissolution and volume variation of materials.^81,82^ For clarity, the application of electrospinning nanofibers in cathode materials is described in the following aspects: vanadium-based materials, manganese-based materials, and other cathode materials.

#### 4.1.1 Vanadium-based cathodes

Vanadium oxides have become one of the most promising cathode materials because of their various oxidation states, high theoretical specific capacity, and abundant crystal structure.^83,84^ However, vanadium-based cathodes will dissolve in mild acidic aqueous electrolytes because of the strong polarity of water molecules and anions, resulting in capacity fading. In addition, dissolved substances will deposit on the surface of the Zn anode, reducing the reactivity and utilization of the Zn metal.^85^ Usually, vanadium-based materials are semiconductors that possess poor electronic conductivity, so highly conductive substances are often used in the preparation of the cathode electrodes to improve the conductivity of the materials.^86^

**Fig. 2:**
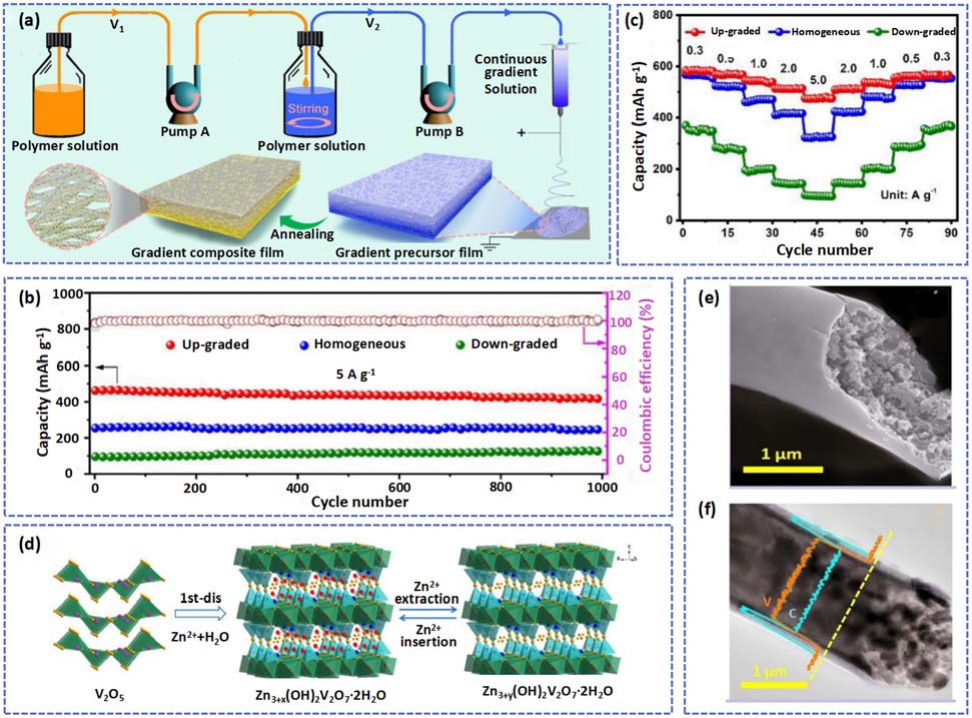
(a) Schematic illustration of fabricating gradient composite films. (b) Cycling performances and (c) rate capabilities of the up-graded cathode. Adapted from ref. 90, copyright 2022, Elsevier B.V. (d) Schematic illustration of the reaction mechanism of the V_2_O_5_ electrode. Adapted from ref. 38, copyright 2019, Elsevier B.V. (e) SEM and (f) TEM images of the hierarchical hybrid fibers with V_2_O_5_. Adapted from ref. 91, copyright 2019, American Chemical Society.

To alleviate these limitations, numerous approaches have been proposed to enhance the electrochemical performance of vanadium-based materials. Among them is preparing V_*x*_O_*y*_ nanofibers by the electrospinning technique with excellent ion diffusion pathways, high conductivity, and nanostructures, which promote electron/ion transport and improve the cycling ability of the cathode. For example, to address the problems of dissolution and poor conductivity of VO_2_, Liu *et al.* prepared self-supported VOC-NF composites by the electrospinning method followed by steam treatment, in which VO_2_ nanodots were embedded in carbon nanowires.^87^ In VOC-NF, the carbon shell with good electrical conductivity not only prevented the dissolution of the vanadium element but also avoided the use of binder and conductive species, resulting in high discharge specific capacity and energy density.^88^ Therefore, the vanadium-based cathode exhibited a satisfactory electrochemical performance due to the rapid Zn^2+^ diffusion and electron transfer. Generally speaking, the component distribution of the polymer solution determines the content and distribution of active materials in electrospun nanofibers.^89^ The concentration distribution of precursors during the electrospinning process could therefore be adjusted to produce nanofibers with a continuous concentration gradient. Niu's group combined a dynamic concentration adjustment technique and electrospinning method to develop continuous gradient composite films (GCFs) (Fig. 2a).^90^ The polymer solution was continuously added to the precursor solution to form a continuously diluted resultant precursor solution. In VO-GCFs, VO nanoparticles were gradient distributed throughout the carbon fiber matrix after the electrospinning and annealing process. In particular, the electronic conductivity of VO-GCFs gradually increased with the gradient distribution of VO nanoparticles, which facilitated the rapid transfer of electrons and improved the reaction kinetics and electrochemical performance. Compared with homogeneous or down-graded VO-GCFs, the up-graded cathode exhibited an excellent cycling and rate ability. Hence, at a current density of 5.0 A g^−1^, the discharge capacity of the Zn//VO-GCFs battery was nearly unchanged after 1000 cycles (Fig. 2b). In the rate performance test, the average discharge capacity of the up-graded cathode was 477.1 mA h g^−1^ at 5 A g^−1^. As the current density became 0.3 A g^−1^, the capacity retention of the up-graded cathode reached 81.2% (Fig. 2c).

Constructing a microstructure can efficiently improve the transport kinetics of cathode materials.^12^ For instance, a hierarchical structure could shorten ion transport pathways,^92,93^ a porous structure with a large SSA can provide abundant transfer channels for Zn^2+^,^85^ a hollow structure can act as a host to load active materials,^94^*etc.* Some researchers have prepared many vanadium-based nanofibers with special structures to improve the cycling ability of electrodes. For example, Chen *et al.* successfully produced porous V_2_O_5_ nanofibers *via* the electrospinning method followed by calcination.^38^ This abundant mesoporous structure is conducive to electrolyte permeation and Zn^2+^ insertion. In the first charging process, the V_2_O_5_ transformed into Zn pyrovanadate with a highly stable open framework, which greatly favors the reversible Zn^2+^ insertion/extraction (Fig. 2d). Therefore, after 500 cycles, the battery with a V_2_O_5_ nanofiber cathode showed a high capacity of 166 mA h g^−1^ and an impressive capacity retention of 81% at 2C. Furthermore, Wang *et al.* fabricated novel hybrid fibers with core/shell hybrid fibers (Fig. 2e and f), which promoted rapid electron/ion transmission and high mass loading, thus gaining a better energy storage capability and rate performance ability.^91^

**Fig. 3:**
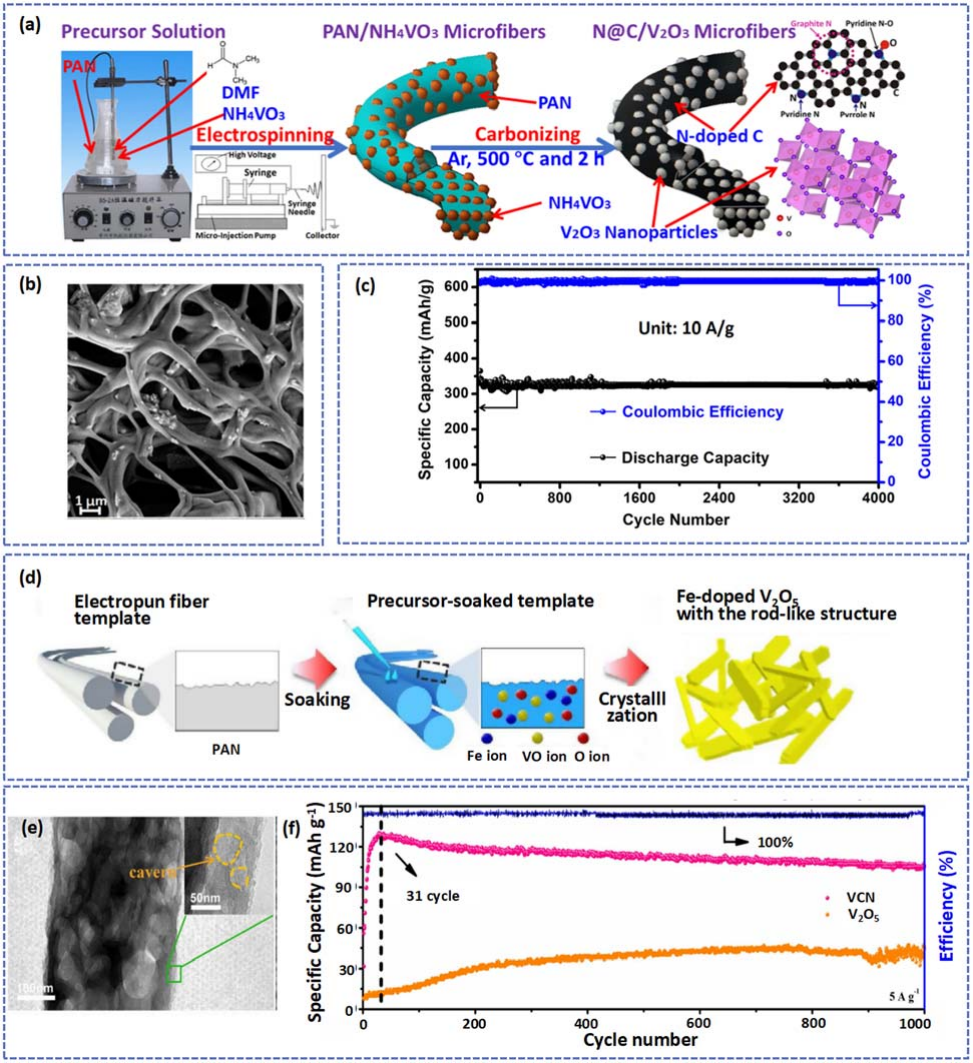
(a) A diagrammatic representation of the synthetic procedure and structure of N@C/V_2_O_3_ composites. (b) SEM image of samples. (c) The cycling ability and coulombic efficiency of the N@C/V_2_O_3_ cathode at 10 A g^−1^. Adapted from ref. 98, copyright 2020, Elsevier B.V. (d) An illustration of the synthetic process for Fe-doped V_2_O_5_. Adapted from ref. 99, copyright 2021, Elsevier B.V. (e) TEM image of VCN fibers. (f) Long-term cycling performance of VCN at 5 A g^−1^. Adapted from ref. 75, copyright 2020, Elsevier B.V.

Heteroatom doping is an effective method to modify the intrinsic electronic/ionic properties of electrode materials for AZIBs.^95^ Doped heteroatoms can widen the interlamellar spacing and redistribute the charge of the surface atoms, increasing ion storage and facilitating electron transport.^96,97^ During the process of electrospinning, N-containing polymers (such as PVP and PAN) were often used. These polymers were transformed into N-doped carbon nanofibers after carbonization, which contributed to an increase in the electronic conductivity of materials and provided more active sites for Zn^2+^ insertion/extraction. For instance, Zhang *et al.* fabricated an N-doped VN-encapsulated carbon nanofiber (VN/N-CNFs) compound by carbonizing H_2_BDC and VCl_3_/PAN fibers.^76^ The 3D self-supported hierarchical structure of the VN/N-CNFs process was thus directly applicable as an electrode for AZIBs and exhibited ultra-long cycle lifetimes and super-high rates. As shown in Fig. 3a and b, Zhang *et al.* prepared N-doped C/V_2_O_3_ (N@C/V_2_O_3_) microfibers by the electrospinning method.^98^ The graphitic N atoms in the composites could promote charge transfer and improve the electrical conductivity and stable cycling ability of N@C/V_2_O_3_. Thus, the battery based on the N@C/V_2_O_3_ electrode delivered a specific capacity of 322.3 mA h g^−1^ and superhigh capacity retention of 91.7% after 4000 cycles at 10 A g^−1^ (Fig. 3c). Besides, Yoo *et al.* produced Fe-doped V_2_O_5_ nanorods by immersing the PAN fiber templates in sol solutions with vanadium salt and iron salt followed by calcination (Fig. 3d).^99^ As an outstanding cathode for AZIBs, the Fe-V_2_O_5_ not only shortened the diffusion distance of Zn^2+^ but also provided extra active sites for Zn^2+^ storage.

Under thermal treatment, carbon will consume the lattice of materials or surface O atoms to form defects.^24^ For example, at high temperatures, vanadium oxide nanofibers (VCN) were generated with physical and chemical defects by decomposing VO(acac)_2_/PAN precursor fibers.^75^ The physical defects such as pore pathways and caverns can provide more storage sites for Zn^2+^ and abundant chemical defects benefit the Zn^2+^ insertion/extraction during cycling (Fig. 3e). As shown in Fig. 3f, compared with V_2_O_5_, the Zn//VCN cell produced higher capacity retention of about 83% and stabler coulombic efficiency (almost 100%) at 5 A g^−1^ after cycling over 1000 cycles, which was attributed to the synergistic effect of dual defects. Table 1 summarizes the electrochemical performances of vanadium-based materials with electrospinning fibers.

**Table 1:** A summary of electrospinning vanadium-based nanofiber materials for AZIBs

Materials	Electrospinning solution (precursor/polymer/solvent)	Structure	Long cycle performance	Rate performance	Ref.
VOC-NF	C_10_H_14_O_5_V/PAN/DMF	—	120 mA h g^−1^ at 20 A g^−1^ after 18 000 cycles (63% capacity retention)	215 mA h g^−1^ at 20 A g^−1^	87
VO-GCFs	VOC_2_O_4_/PAN/DMF	Porous	The capacity is nearly unchanged after 1000 cycles at 5 A g^−1^	477.1 mA h g^−1^ at 5.0 A g^−1^	90
V_2_O_5_-CFC	V_2_O_5_/PAN/DMF	—	154 mA h g^−1^ at 0.5 A g^−1^ after 1000 cycles	91 mA h g^−1^ at 4 A g^−1^	100
V_2_O_5_	VO_2_/PVP/H_2_O_2_	—	The capacity is 36% of the maximum value after 500 cycles at 2 A g^−1^	179 mA h g^−1^ at 2 A g^−1^	101
V_2_O_5_	NH_4_VO_3_, H_2_C_2_O_4_·2H_2_O/PVP/DMF	Porous	166 mA h g^−1^ at 2C after 500 cycles (81% capacity retention)	104 mA h g^−1^ at 10C	38
V_2_O_5_/Zn_2_V_2_O_7_	C_15_H_21_O_6_V, C_2_H_4_O_2_, PMMA/PAN/DMF (NH_4_VO_3_, Zn(NO_3_)_2_, H_2_O, PMMA/PAN/DMF)	Hierarchical	High capacity retention (for V_2_O_5_ it is 95.8% and for Zn_2_V_2_O_7_ it is 93.1%) after 8000 cycles at 8 A g^−1^	—	91
VN/N-CNFs	H_2_BDC, VCl_3_/PAN/DMF	Hierarchical	482 mA h g^−1^ at 50 A g^−1^ after 30 000 cycles	297 mA h g^−1^ at 100 A g^−1^	76
N@C/V_2_O_3_	NH_4_VO_3_/PAN/DMF	—	322.3 mA h g^−1^ at 10 A g^−1^ after 4000 cycles (91.7% capacity retention)	242.2 mA h g^−1^ at 30 A g^−1^	98
Al_2_O_3_@VSe_2_ NSs@N-CNFs	VO(acac)_2_/PAN, PVP/DMF	Core/shell	502.2 mA h g^−1^ at 0.05 A g^−1^ after 500 cycles (91.6% capacity retention)	—	43
V_2_O_3_@C NFs	C_15_H_21_O_6_V/PAN/DMF	—	65 mA h g^−1^ at 2 A g^−1^ after 1000 cycles	100 mA h g^−1^ at 2 A g^−1^	102
Fe-doped V_2_O_5_	PAN/DMF	—	The capacity retention is 85% after 160 cycles at 1.3 A g^−1^	256 mA h g^−1^ at 2 A g^−1^	99
VCN	VO(acac)_2_/PAN/DMF	Hierarchical	1000 cycles (83% capacity retention)	73 mA h g^−1^ at 10 A g^−1^	75
V_2_O_5_	PAN/DMF	—	The capacity retention is 74.6% after 300 cycles at 1.3 A g^−1^	—	103

#### 4.1.2 Manganese-based cathodes

Manganese-based materials, including MnO, MnO_2_, Mn_2_O_3_, Mn_3_O_4_, ZnMn_2_O_4_, MnS, and so on, have been widely studied in AZIBs because of their numerous merits such as high operating voltage, cheapness, abundant resources, and nonpoisonous nature.^104^ Unfortunately, some challenges prevent its practical application.^105^ Manganese-based electrodes are usually constructed of active powder, conductivity agents, binders, and collectors. However, the poor electrical conductivity and random aggregation of manganese-based composites cannot realize fast charging at high current densities.^23^

**Fig. 4:**
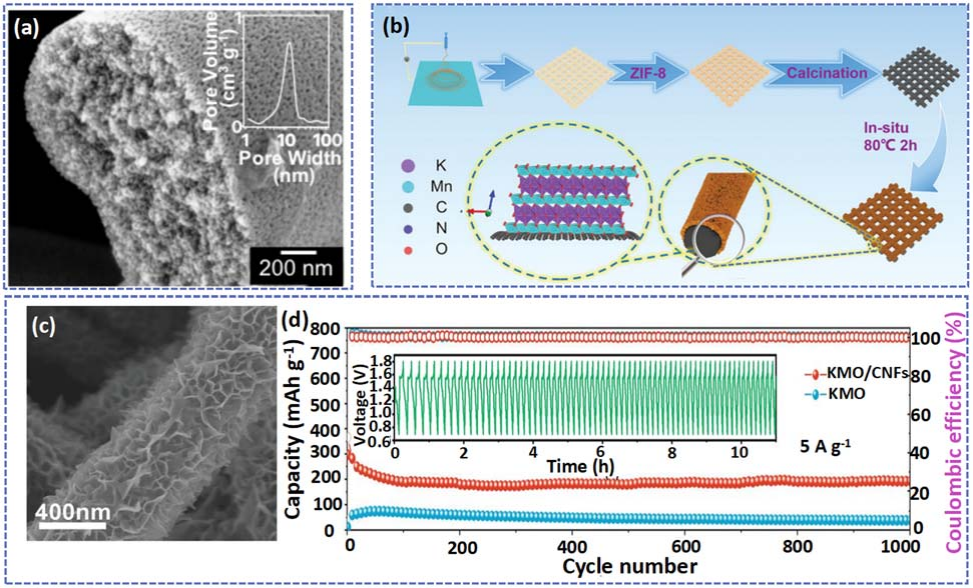
(a) Cross-sectional SEM image of a porous carbon fiber. Adapted from ref. 68, copyright 2022, Wiley-VCH. (b) The fabrication of KMO/CNFs is illustrated schematically. (c) SEM image of KMO/CNFs. (d) Long-term cycling performance of KMO/CNFs at 3.0 A g^−1^. Adapted from ref. 106, copyright 2022, Wiley-VCH.

Carbon nanofibers with large SSA and high electrical conductivity can be used as conductive substrates for cathode materials loading, which not only facilitates fast electron transfer but also simply the preparation of electrodes without binders and conductive additives.^44^ For example, Guo *et al.* used porous carbon fibers (PCF) to support MnO_2_ to form a free-standing PFC@MnO_2_ electrode.^68^ Specifically, the graphitic PCF fabricated by the electrospinning technique and high-temperature treatment with high electrical conductivity and uniform pores (Fig. 4a) favors the mass loading of MnO_2_ (59.1%) and fast charging. As a result, owing to the fast ion/electron transport ability of PFC@MnO_2_, the Zn//PFC@MnO_2_ displayed impressive structural stability at various current densities. Besides, Yang *et al.* prepared high-flexibility nitrogen-doped carbon films through an electrospinning technique and calcination with PAN, PVP, 2-methylimidazole, and zinc acetate as raw materials (Fig. 4b).^106^ During carbonization, the evaporation of Zn endowed the CNFs with a porous structure, which not only provided abundant reaction sites for the growth of δ-MnO_2_ but also had a strong electrostatic attraction for Mn^2+^. As displayed in Fig. 4c, the lamellar-like K^+^-intercalated δ-MnO_2_ (KMO) was loaded on the surface of CNFs *via* the hydrothermal method of KMnO_4_, and the resulting KMO/CNFs presented a large surface area to enable expansion of the contact area between KMO and the electrolyte and promote ion transfer. Therefore, even after 1000 cycles at 3 A g^−1^, the KMO/CNFs still exhibited a reversible capacity of 190 mA h g^−1^ (Fig. 4d). What's more, compared with KMO, KMO/CNF showed lower charge-transfer and ion-diffusion kinetics, which was attributed to the existence of CNFs. Hiralal *et al.* explored the relationship between the capacity of the battery and the diameter of carbon fibers when carbon fibers were used as the substrate for the cathode.^37^ The results showed that decreasing the diameter will enhance the surface area, charge collection area, and conductivity of carbon fibers, which will promote electrolyte diffusion in the electrode, resulting in a higher capacity battery.

There is no doubt that using carbon nanofibers as a substrate is an effective way to improve the electrical conductivity of manganese-based compounds. However, the construction of a firm and tight interface between active materials and carbon fibers is still a great challenge that needs to be addressed in the future.

**Fig. 5:**
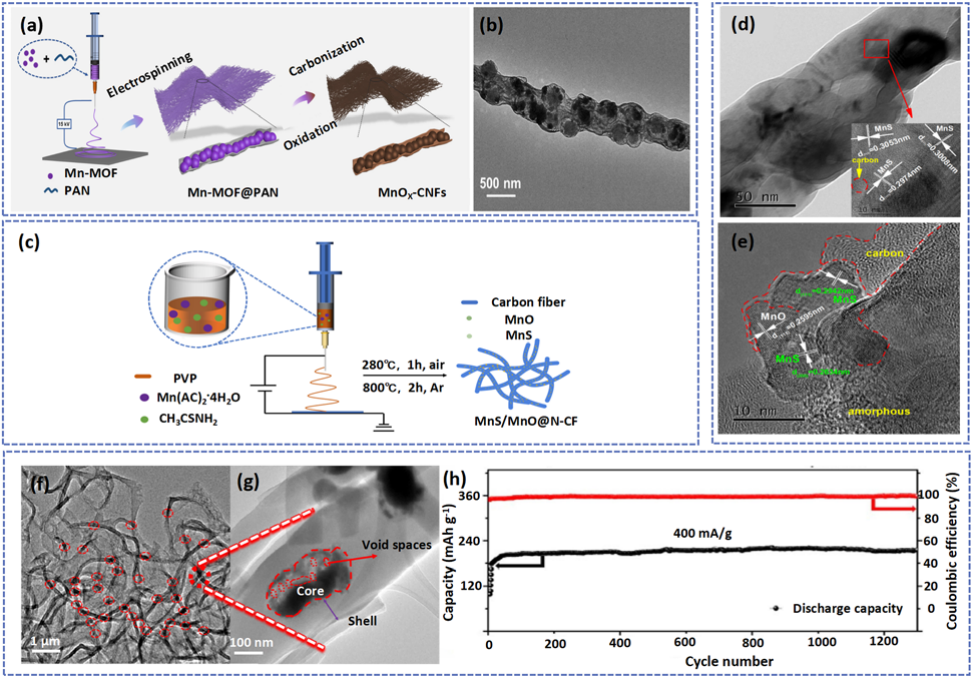
(a) Schematic illustration of the synthesis process and (b) TEM image of MnO_*x*_-CNFs. Adapted from ref. 31, copyright 2022, American Chemical Society. (c) An illustration of the MnS/MnO@N-CF synthesis process. (d) SEM and (e) TEM images of MnS/MnO@N-CF. Adapted from ref. 27, copyright 2022, Elsevier B.V. (f and g) TEM images of Mn_3_O_4_@HCFs. (h) Long cycling performance and coulombic efficiency of the Mn_3_O_4_@HCF electrode at 0.4 A g^−1^. Adapted from ref. 59, copyright 2020, Elsevier Ltd.

Embedding active substances in carbon nanofiber matrixes could inhibit the dissolution of manganese-based materials and construct highways for electrons.^32,107,108^ For instance, Ding *et al.* prepared CNF coated bead-like manganese oxide (MnO_*x*_-CNFs) *via* the electrospinning method (Fig. 5a).^36^ As shown in Fig. 5b, the MnO_*x*_ particles were tightly encapsulated in the amorphous carbon layer, which effectively relieved its dissolution. Moreover, Wu's group embedded MnS/MnO with the heterostructures in N-doped carbon fibers to form MnS/MnO@N-CF with high ion and electron conductivity (Fig. 5c).^32^ As shown in Fig. 5d and e, the MnS/MnS nanoparticles were uniformly dispersed in carbon matrixes, and the edges of active materials were connected by a large amount of amorphous carbon, which was conducive to the storage of electrolyte and the enhancement of the conductivity of the materials. Benefiting from the protection of the carbon layer, the structure of active materials remained stable without collapse and pulverization after cycling, indicating an excellent stable cycling ability of the electrode.

However, this strategy will partly reduce the ion transport efficiency and active substance utilization of active materials. As a result, the precise control of the structure of nanofibers is essential to achieve a cathode with excellent electrochemical performance. As a typical example, Long *et al.* fabricated Mn_3_O_4_@HCFs with core/shell structure by a coaxial electrospinning method and subsequent high temperature treatment.^63^ This fiber consisted of a carbon shell with a thickness of about 70 nm (content of 12.7 wt%) and Mn_3_O_4_ nanoparticles (Fig. 5f and g). The amorphous carbon layer not only served as a protective layer between Mn_3_O_4_ and the electrolyte, preventing the dissolution of the active substance, but also mitigated the volume expansion of the electrode during cycling. In addition, the void spaces between the carbon shell and the Mn_3_O_4_ core can accommodate a large amount of electrolyte, providing space for electrochemical reactions. Therefore, the battery based on the Mn_3_O_4_@HCFs cathode material displayed ultra-stable cycling capability with 96.9% capacity retention and high coulombic efficiency of around 100% after 1300 cycles at 0.4 A g^−1^ (Fig. 5h). The precise control of nano- and microstructures can also be achieved by template methods.^109^ For example, the manganese dioxide precursor was wrapped on the surface of a CNF matrix using a hydrothermal method and then calcining to obtain tunnel-structured α-K_0.19_MnO_2_ nanotubes.^110^ It is worth noting that the CNF as the template will be consumed during the calcining process. Owing to the stability of the structure of α-K_0.19_MnO_2_, the cathode possessed excellent rate and cycling performance. Table 2 summarizes the electrochemical performances of manganese-based materials with electrospinning fibers.

**Table 2:** A summary of electrospinning manganese-based nanofiber materials for AZIBs

Materials	Electrospinning solution (precursor/polymer/solvent)	Structure	Long cycle performance	Rate performance	Ref.
PFC@MnO_2_	PMMA-*b*-PAN/DMF	Porous	A high capacity of 184 mA h g^−1^ at 1 A g^−1^	—	68
KMO/CNFs	Zn(AC)_2_/PVP, PAN/DMF	Porous	190 mA h g^−1^ at 3 A g^−1^ after 1000 cycles	236 mA h g^−1^ at 3 A g^−1^	106
δ-MnO_2_-CNFs	BTDA, BZD/PVP, PAA/DMF	—	120.9 mA h g^−1^ at 1 A g^−1^ after 500 cycles	127.3 mA h g^−1^ at 2 A g^−1^	111
V_O_-MnO_2_@CNFs	PAN/DMF	—	135 mA h g^−1^ at 1 A g^−1^ after 740 cycles	148 mA h g^−1^ at 1 A g^−1^	44
MnS/MnO@N-CF	Mn(Ac)_2_, C_2_H_5_NS/PVP/ethanol	—	151 mA h g^−1^ at 0.5 A g^−1^ after 400 cycles	128.7 mA h g^−1^ at 2 A g^−1^	32
MnO@N-C	Mn(Ac)_2_/PVP/ethanol	—	176.3 mA h g^−1^ at 0.5 A g^−1^ after 200 cycles	66.3 mA h g^−1^ at 2 A g^−1^	107
MnO_1−*x*_@CNF	Mn(Ac)_2_/PAN/DMF	—	96 mA h g^−1^ at 2 A g^−1^ after 2500 cycles (90% capacity retention)	158 mA h g^−1^ at 1 A g^−1^	108
MnO_*x*_-CNFs	Mn-MOF/PAN/DMF	Bead-like	The capacity retention is 71% after 5000 cycles at 3 A g^−1^	131.4 mA h g^−1^ at 5 A g^−1^	36
Mn_3_O_4_@HCFs	Mn_3_O_4_/PAN/DMF	Core/shell	The capacity retention is 96.9% after 1300 cycles at 0.4 A g^−1^	115.7 mA h g^−1^ at 2 A g^−1^	63
α-K_0.19_MnO_2_	PAN/DMF	—	211 mA h g^−1^ at 1C after 2500 cycles (78% capacity retention)	113 mA h g^−1^ at 20C	110
Mn_3_O_4_	Mn(Ac)_2_/PVP/H_2_O, ethanol	—	104 mA h g^−1^ at 2 A g^−1^ after 1000 cycles	153 mA h g^−1^ at 5 A g^−1^	112

#### 4.1.3 Other cathode materials

**Fig. 6:**
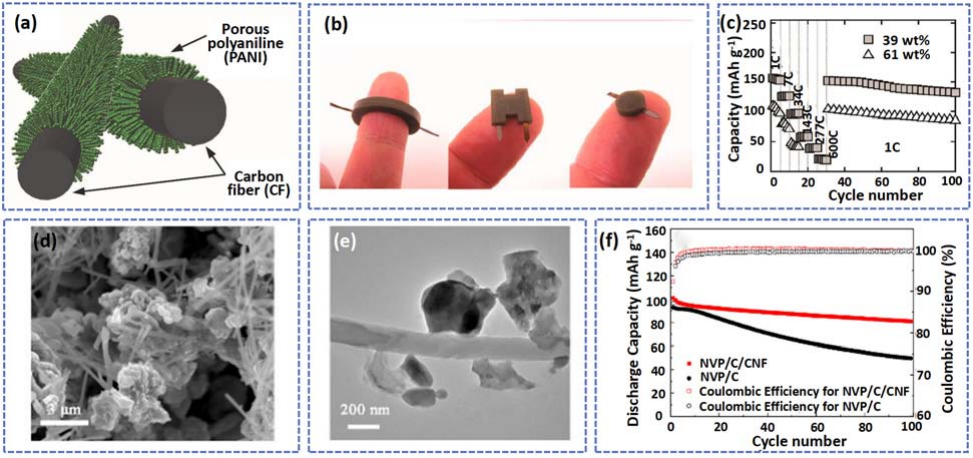
(a) A schematic diagram showing the *in situ* polymerization of aniline in an aqueous solution to synthesize a PANI/CF cathode. (b) Optical images of ring-, H-, and cylindrical shapes of Zn-PANI batteries. (c) Cycling ability of the cells with different PANI loading. Adapted from ref. 34, copyright 2018, American Chemical Society. (d) SEM and (e) TEM images of NVP/C/CNF. (f) Cycle performance of NVP/C/CNF and NVP/C at 0.1 A g^−1^. Adapted from ref. 109, copyright 2021, American Chemical Society.

In addition to vanadium-based and manganese-based materials, many other cathode materials were prepared by the electrospinning method. Kim *et al.* fabricated a freestanding carbon fiber (CF) as a current collector to support polyaniline (PANI) *via* electrospinning and carbonization (Fig. 6a).^39^ Especially, the CF with high conductivity (resistance about 20 Ω sq^−1^) was firstly activated by HNO_3_ treatment to increase the number of active sites (some groups such as C=O, C–O, and O–C=O), which can promote the *in situ* polymerization of aniline monomers on the CF surface to achieve a PANI/CF cathode. Due to the high conductivity of the 3D CF, the PANI/CF showed a small electron resistance of about 400 Ω sq^−1^, allowing the fast transfer of electrons. Benefiting from the high conductivity and free-standing structure of composites, the PANI/CF can be used as an electrode directly without binder and conductive additives to assemble batteries in arbitrary geometries (Fig. 6b). As displayed in Fig. 6c, the battery with the PANI/CF electrode delivered excellent rate performance at 600C.

Xu *et al.* synthesized a composite in which hybrid carbon coated Na_3_V_2_(PO_4_)_3_ was interconnected with carbon nanofibers (NVP/C/CNF) by electrospinning and sol–gel methods.^113^ As displayed in Fig. 6d and e, the NVP nanoparticles were randomly wrapped tightly in CNF to form a 3D conductive network to improve the electron conductivity and stable structure ability of the composite. Compared to NVP/C, the NVP/C/CNF electrode exhibited a more stable cycling ability. The battery based on NVP/C/CNF displayed a high capacity retention of 82.5% after 100 cycles at 0.1 A g^−1^, which is much higher than that of the battery based on NVP/C (52.7%) (Fig. 6f). A comparison of the performance of other cathode materials is presented in Table 3.

**Table 3:** A summary of electrospinning nanofibers for other cathode materials of AZIBs

Materials	Electrospinning solution (precursor/polymer/solvent)	Structure	Long cycle performance	Rate performance	Ref.
PANI/CF	PAN/DMF	—	—	The capacity fade was about 20%	39
NVP/C/CNF	—	—	The capacity retention is 82.5% after 100 cycles at 0.1 A g^−1^	65.0 mA h g^−1^ at 1.0 A g^−1^	113

### 4.2 Anodes

In aqueous electrolytes, the thermodynamic and electrochemical instability of the Zn metal anode dramatically shortens the service life of AZIBs and limits their practical applications.^114,115^ Among them, thermodynamic instability is manifested by serious corrosion reactions on the surface of Zn during cycling, which consumes the active Zn and decreases the coulombic efficiency of the Zn anode. The electrochemical instability is presented by uncontrollable dendrite growth, where the formed Zn dendrites will penetrate the separator, ultimately leading to the failure of the cell.^116^ As a result, various approaches have been proposed to address these above issues, including (1) optimizing the composition and concentration of electrolytes to stabilize the Zn anode;^117,118^ (2) protecting the Zn anode surface from direct contact with the electrolyte by forming an interfacial layer and reducing the occurrence of corrosion side reactions;^119,120^ and (3) constructing a 3D substrate that can help reduce local current densities and promote the uniform distribution of Zn^2+^, which is advantageous for the homogeneous deposition of Zn and inhibits the growth of dendrites.^78,121^ Among them, interfacial layer modification and 3D substrate construction are effective and direct strategies to protect the Zn anode. Carbon and polymer fibers fabricated by the electrospinning method with high flexibility adjustable structures are considered to be an ideal material for use as the protective layer and substrate for the Zn anode. Therefore, we will summarize and discuss the application of electrospinning fibers for protective layers and substrates of the Zn anode.

#### 4.2.1 Pure carbon fibers

**Fig. 7:**
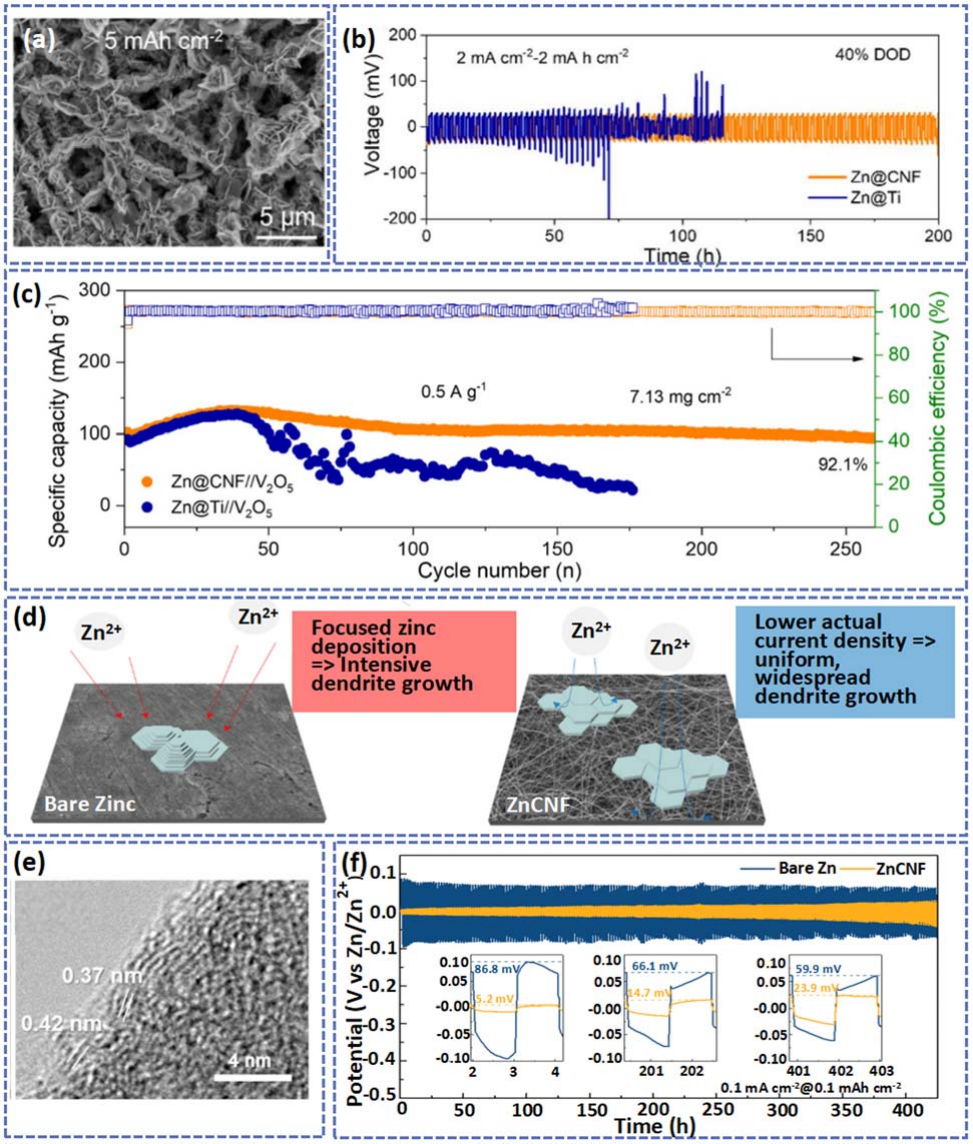
(a) SEM image of CNFs after being electrodeposited with an amount of Zn at a current density of 0.5 mA cm^−2^ with a capacity of 5.0 mA h cm^−2^. (b) Cycling performance of symmetric cells with different electrodes at 2 mA cm^−2^. (c) Cycling ability of full cells at the current density of 0.5 A g^−1^. Adapted from ref. 45, copyright 2022, American Chemical Society. (d) The deposition behaviors of Zn^2+^ on the different substrates. (e) HRTEM pattern of CNF. (f) Long cycling performance of bare Zn and ZnCNF symmetric cells. Adapted from ref. 124, copyright 2022, John Wiley & Sons Ltd.

The unique advantages of carbon materials as a substrate or protective layer for the Zn anode can be summarized in the following aspects: (1) the carbon materials with large SSA and porous structure can lower the local current density and accommodate the volume variation of the Zn anode during cycling. (2) A carbon substrate-based anode with high flexibility and processibility can be used to assemble flexible batteries. (3) A carbon protecting layer can provide abundant ion channels to promote the transfer of Zn^2+^ and inhibit the formation of Zn dendrites. As a typical carbon material, carbon fibers exhibit high axial strength, low density, good expansion, anisotropy, and excellent corrosion resistance.^122,123^ In particular, the diameter and porosity of carbon fibers can be controlled by the electrospinning method, which has more practical applications in anodes.^124^ For example, carbon nanofiber frameworks were prepared by electrospinning and calcination treatments, where the diameter (about 200 nm) and porosity of the nanofibers could be adjusted by electrospinning parameters.^45^ Interestingly, the plasma treatment improved the surface hydrophilicity of the carbon fibers, which was conducive to promoting the uniform deposition of Zn^2+^. Thus, benefiting from the coordination of the 3D framework, conductivity, and hydrophilicity of the carbon fibers, Zn was homogeneously deposited on the carbon fibers without severe aggregation at a current density of 0.5 mA cm^−2^ with an areal capacity of 5 mA h cm^−2^ (Fig. 7a). Most importantly, at a 40% depth of discharge (DOD) (an areal capacity of 2 mA h cm^−2^), the Zn@CNF‖Zn@CNF symmetric cell was stably cycled over 193 h at a current density of 2 mA cm^−2^ (Fig. 7b). As demonstrated in Fig. 7c, compared with Zn@Ti//V_2_O_5_, the battery of Zn@CNF//V_2_O_5_ displayed a better cycling ability.

In their study, Baek *et al.* produced a ZnCNF anode through the electro-deposition of Zn on the surface of electrospun carbon nanofibers.^124^ The 3D porous network of carbon with large SSA (53.04 m^2^ g^−1^) and high conductivity (830 S m^−1^) can decrease the local current density during the cycling process and provide more nucleation sites, thus reducing the nucleation overpotential of Zn in the initial stage. Meanwhile, the graphitic carbon with a low lattice mismatch interfacial layer to the Zn (002) plane can promote the preferred orientation of Zn to the (002) plane. Consequently, compared with bare Zn, the ZnCNF showed a smooth and compact anode surface after cycles (Fig. 7d and e). As shown in Fig. 7f, the symmetric cell demonstrated a stabler plating/stripping behavior with a small voltage hysteresis of 23.9 mV after 400 cycles at the current density of 0.1 mA cm^−2^ with an areal capacity of 0.1 mA h cm^−2^.

#### 4.2.2 Carbon fibers with zincophilic materials

Although the pure carbon fibers with large SSA can contribute to the homogeneous distribution of the electric field and confine the Zn in 3D pores to avoid its accumulation during the stripping/plating processes, the hydrophobic and zincophobic carbon matrixes lead to a high energy barrier of Zn nucleation, which is unfavorable for the uniform growth of Zn.^125^ The nucleation behavior of Zn is greatly affected by the surface properties of the substrate. Herein, zincophilic materials (such as functional groups and metal nanoparticles) are introduced on the surface of carbon matrixes to reduce nucleation polarization, achieving a highly reversible Zn cycling process and inhibiting the formation of Zn dendrites.

**Fig. 8:**
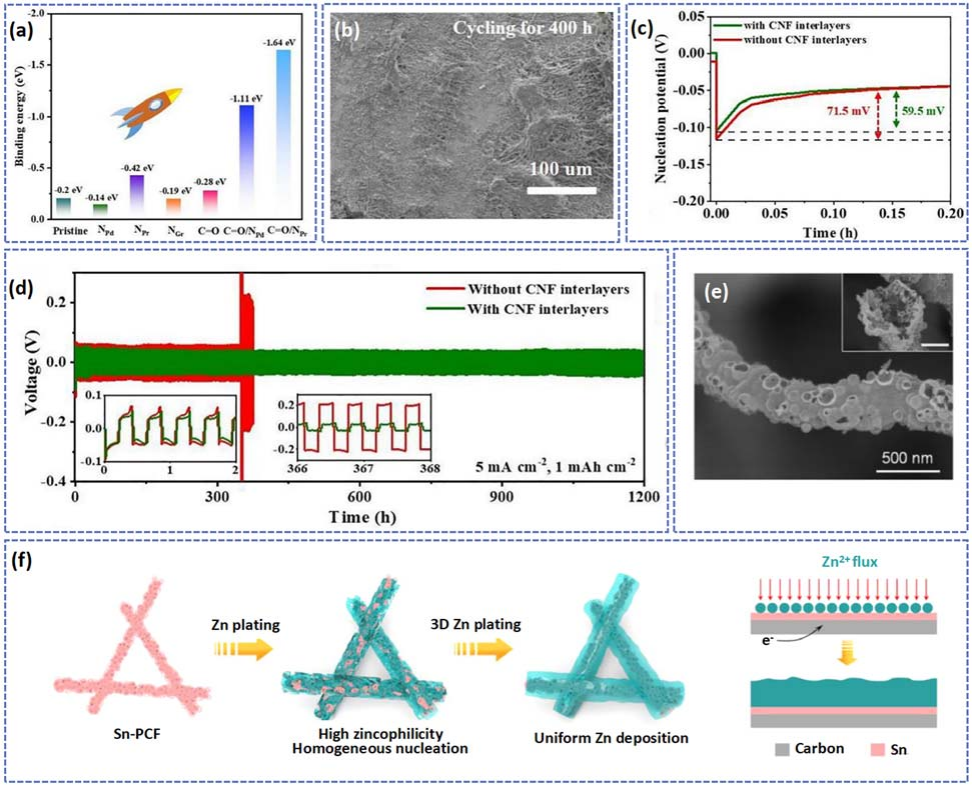
(a) A comparison of the binding energy between Zn atoms and different adsorption sites. The morphology (b), charge/discharge curves (d), and Zn nucleation overpotential (c) at 5 mA cm^−2^ with a capacity of 1 mA h cm^−2^. Adapted from ref. 33, copyright 2021, Elsevier B.V. (e) The SEM image and (f) Zn plating and nucleation diagrams on Sn-PCF. Adapted from ref. 134, copyright 2022, Elsevier B.V.

The functional groups including N,^125,126^ C=O,^127^ F,^128^ and –NH_2_ (ref. 129) with high electronegativity serve as zincophilic sites to capture the positively charged Zn^2+^, guiding the homogeneous nucleation and plating of Zn. Chen's group fabricated a 3D N-doped carbon nanofiber film@Zn (3DN-C@Zn) anode to assemble a 3DN-C@Zn//AlVO-DMF battery. The N doping can improve the hydrophilicity of carbon fibers, decreasing the diffusion energy barrier of Zn^2+^.^130^ Therefore, the 3DN-C@Zn//AlVO-DMF battery was stably cycled over 200 cycles at 1 A g^−1^ without obvious capacity decay, which is better than that of bare Zn which suffered a short circuit after three cycles at the same current density. Besides, Zhang's group reported a novel N,O co-doped carbon nanofiber interlayer of a Zn anode *via* the electrospinning method combined with carbonization treatment.^33^ At high temperatures, the PAN fibrous membrane transformed into a freestanding carbon fiber interlayer doped with abundant O and N atoms. As the result of theoretical calculation, compared with other sites, the C=O/N_Pd_ (−1.11 eV) and C=O/N_Pr_ dual doping sites (−1.64 eV) showed higher binding energy with the Zn atom, indicating a higher ability to absorb Zn^2+^ (Fig. 8a). Therefore, owing to the porous structure of carbon fibers and high Zn affinity of N and O heteroatoms, a compact and flat Zn deposition layer on the carbon fiber interlayer can be observed after cycling for 400 h at 5 mA cm^−2^ (Fig. 8b). As exhibited in Fig. 8c and d, at the current density of 5 mA cm^−2^ and areal capacity of 1 mA h cm^−2^, the modified symmetric cell displayed a lower nucleation potential of about 59.5 mV, and a stabler cycling ability (over 1200 h).

In addition, the introduction of zincophilic metal nanoparticles such as Ag,^131^ Sn,^121^ Co,^132^ and In^133^ on the substrate can also enhance the zincophilicity of the carbon nanofiber matrix. These zincophilic metal nanoparticles can be coupled with the carbon fibers to stabilize the Zn anode by lowering the nucleation potential of Zn and uniformizing the current density. Yang *et al.* prepared an Sn modified porous carbon fiber (Sn-PCF) framework with a hollow structure to uniformize the deposition of Zn^2+^ (Fig. 8e).^134^ At a high current density of 10 mA cm^−2^ with an areal capacity of 5 mA h cm^−2^, the Sn-PCF@Zn‖Sn-PCF@Zn symmetric cell exhibited a small voltage hysteresis of 47 mV and a long cycle life (over 500 h), which was almost 10 times that of PCF@Zn. In addition, at a current density of 10 A g^−1^, Sn-PCF@Zn//Na_2_V_6_O_16_·1.63H_2_O demonstrated a high capacity retention of 73.5% after 2500 cycles. The reason for the high stable cycle performance of Sn-PCF can be described as the metal Sn possessing a high adsorption ability, which is favorable for regulating the nucleation and deposition of Zn. Besides, the metal Sn can increase the hydrogen evolution energy barrier of the electrode, inhibiting the occurrence of hydrogen evolution reactions. Therefore, owing to the synergetic effect of multifunctional Sn metal and 3D porous carbon, the Zn can be uniformly deposited on the surface of the Sn-PCF (Fig. 8f), and the Sn-PCF@Zn anode had an excellent cycling ability during the test.

Moreover, introducing Cu nanoparticles on the surface of carbon not only improves the conductivity of carbon fibers but also promotes the deposition of Zn. Yang *et al.* reported Cu nanoparticle modified carbon fibers (Cu@CNFs) as the protective layer to stabilize the anode.^135^ Benefiting from the large SSA of carbon fibers and the zincophilicity of Cu nanoparticles, the Cu@CNFs-Zn exhibited low polarization and high deposition/dissolution efficiency in cycling.

**Fig. 9:**
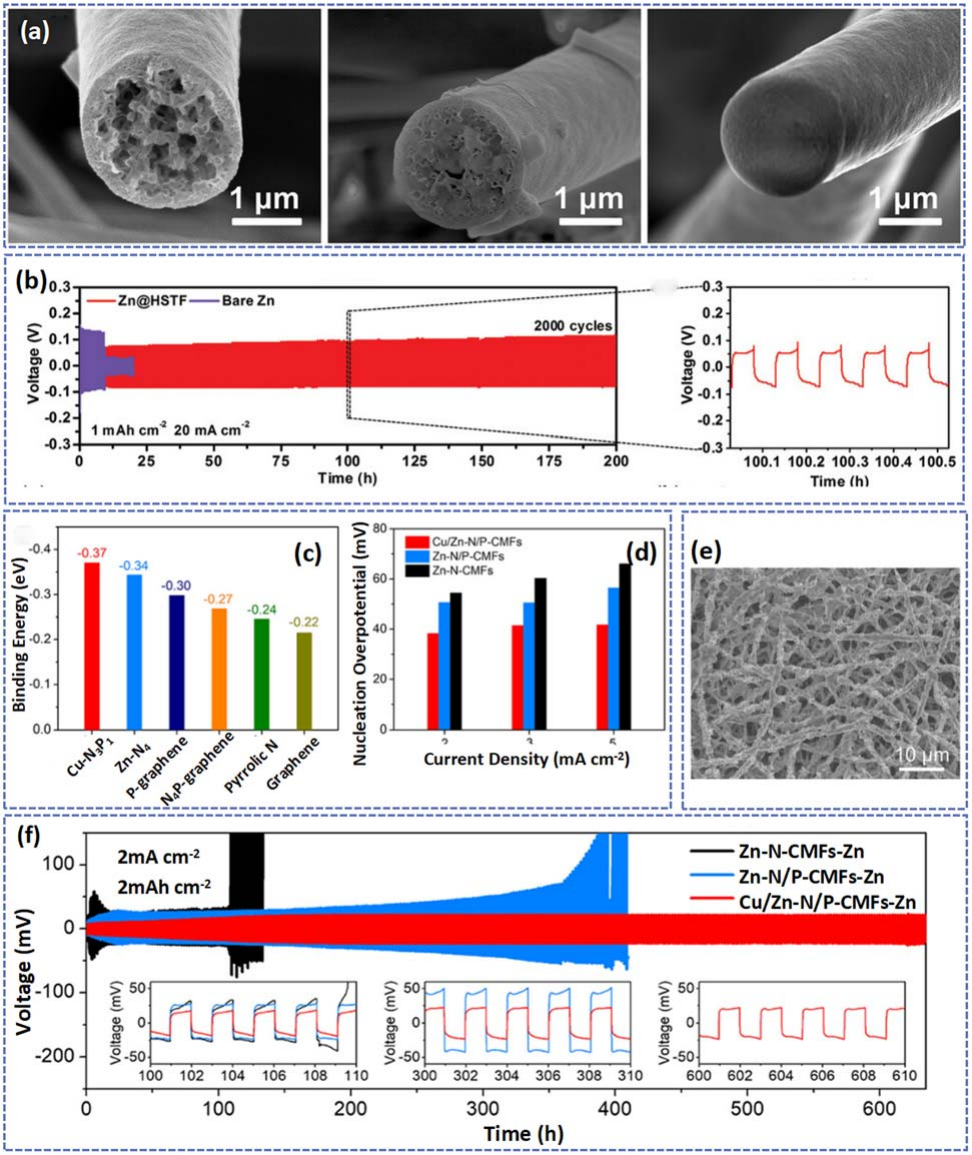
(a) SEM images showing the top view and cross-sections of the HSTF host after plating with various deposition capacities. (b) Voltage profiles of symmetrical cells at current densities of 20 mA cm^−2^ and 1 mA h cm^−2^. Adapted from ref. 35, copyright 2021, Wiley-VCH. (c) A comparison of the binding energy between Zn atoms and different adsorption sites. (d) Nucleation overpotential of Zn on different substrates at current densities of 2, 3, and 5 mA cm^−2^. (e) FESEM image of the Cu/Zn-N/P-CMF framework after Zn plating with capacities of 2 mA h cm^−2^. (f) Cycling performance at 2 mA cm^−2^ and 2 mA h cm^−2^ for symmetric cells using different composite Zn electrodes. Adapted from ref. 42, copyright 2023, American Chemical Society.

In addition to doping metal nanoparticles on carbon fibers to homogenize Zn^2+^ deposition, many researchers have added metal oxides to electrode materials to achieve stable cycling of the Zn anode. For instance, defective ZnO_*x*_ nanoparticles also demonstrated good affinity for Zn, which can be used to enhance the zincophilicity of electrospun carbon fibers.^136^ Xue *et al.* fabricated a 3D porous fiber with TiO_2_ and SiO_2_ uniformly distributed in the interior of hollow HSTF.^35^ Directed by the uniform TiO_2_, the Zn preferred to deposit at the zincophilic TiO_2_ seeds inside the fibers and was further accommodated in the porous carbon fiber matrixes without the growth of Zn dendrites. As shown in Fig. 9a, with the increase in plating capacity, the Zn tended to form a uniform and dense deposition layer in the porous pores rather than the surface of carbon fibers. Besides, the inert material of SiO_2_ can significantly reduce the desolvation active energy during cycling and improve the deposition efficiency of Zn. Consequently, at a high current of 20 mA cm^−2^, the Zn@HSTF anode demonstrated a highly stable plating/stripping behavior over 2000 cycles (Fig. 9b). Furthermore, the Zn@HSTF//MnO_2_ full battery delivered impressive cyclability with 85% capacity retention after 1000 cycles at 1 A g^−1^.

3D carbon fibers with functional groups and metal-based nanoparticles could combine the synergistic effects of two zincophilic materials to homogenize the deposition of Zn^2+^. Yu *et al.* fabricated a 3D conductive fiber network (Sn@NHCF) consisting of N-doped hollow carbon and Sn nanoparticles.^58^ The Sn nanoparticles and doped N element possess high zincophilicity and can reduce the nucleation barrier in cycling. Therefore, even after 100 cycles, the Sn@NHCF-Zn electrode exhibited a high coulombic efficiency of 99.7% at a current density of 5 mA cm^−2^ with 5 mA h cm^−2^. Typically, Zeng *et al.* prepared N,P-codoped carbon macroporous fibers embedded with atomically dispersed Cu and Zn atoms (Cu/Zn-N/P-CMFs) as the host for the deposition of Zn.^42^ It is worth noting that the introduction of N and P atoms not only enhanced the hydrophilicity of carbon fibers but also facilitated the dispersion of Cu and Zn atoms. Besides, they produced Cu-p/Zn-N-CMFs by substituting tannic acid for phytic acid, highlighting the crucial function of P. The results showed that in the absence of PA, Cu aggregated from nanoparticles, which will decrease the reversibility of Zn plating/stripping. The results of theoretical calculation further revealed the zincophilicity of Cu, Zn, N, and P atoms, which can decrease the nucleation overpotential of Zn and favor the oriented deposition of the Zn(002) plane to achieve a dendrite-free anode (Fig. 9c and d). As displayed in Fig. 9e, at a plating capacity of 2 mA h cm^−2^, the Zn was uniformly deposited on the surface of the substrate with parallel nanoflakes. As a result, the Cu/Zn-N/P-CMFs-Zn‖Cu/Zn-N/P-CMFs-Zn cell displayed a small voltage hysteresis (44.9 mV) and a long cycle life (630 h) at a current density of 2 mA cm^−2^ with 2 mA h cm^−2^ (Fig. 9f). In contrast, the battery based on the Zn-N-CMFs-Zn electrode suffered a short-circuit after 110 h due to the serious Zn dendrite growth. Moreover, the Cu/Zn-N/P-CMFs-Zn//MnO_2_ exhibited ultralong life up to 2500 cycles with a capacity retention of 88.8% at 1 A g^−1^.

#### 4.2.3 Polymer fibers

**Fig. 10:**
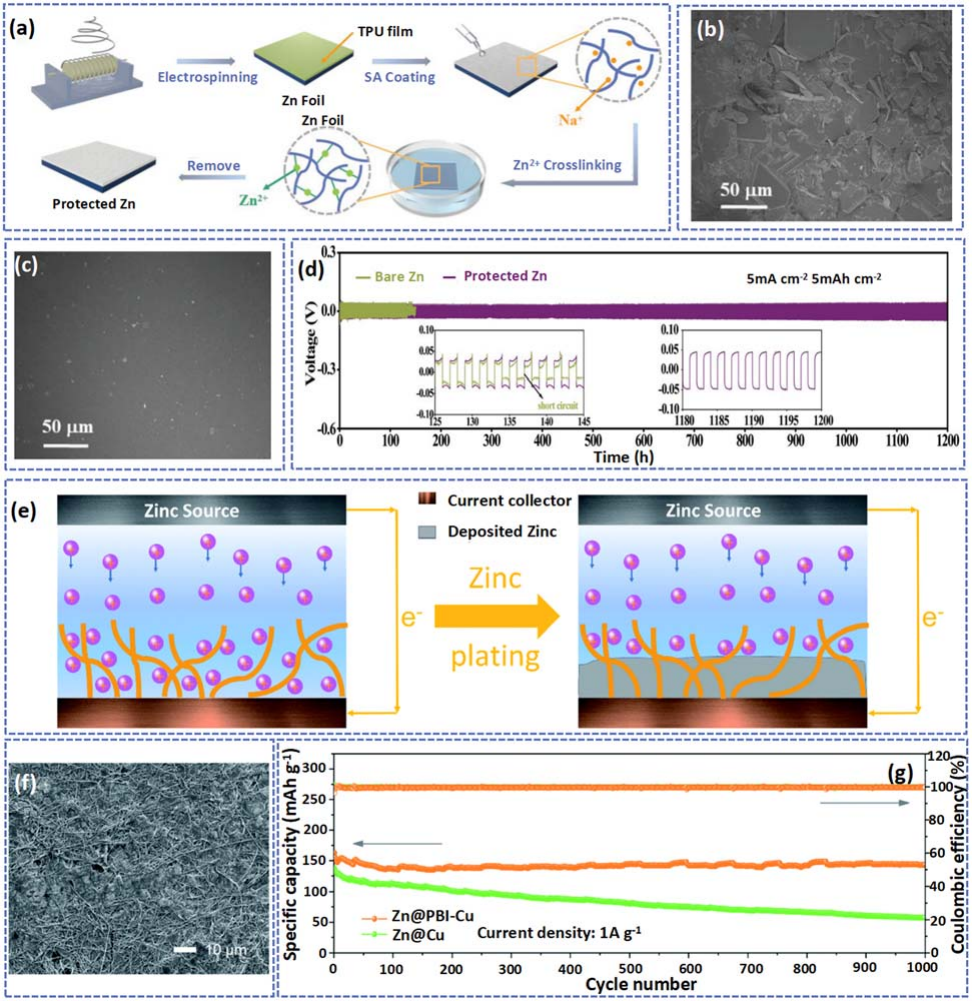
(a) A description of the fabrication process of Zn@TPZA. SEM images of (b) bare Zn and (c) Zn@TPZA after immersion in 2 M ZnSO_4_ electrolyte for 30 days. (d) Cycling performance of bare Zn and Zn@TPZA anodes at 5 mA cm^−2^/5 mA h cm^−2^. Adapted from ref. 133, copyright 2022, Wiley-VCH. (e) Schematic illustration of Zn deposition on a PBI nanofiber framework modified Cu electrode. (f) SEM image of Zn@PBI-Cu after 100 cycles at 10 mA cm^−2^. (g) Long-term cycling performance of the battery at 1 A g^−1^. Adapted from ref. 42, copyright 2020, Royal Society of Chemistry.

Although the excellent conductivity of carbon fibers can reduce charge accumulation and facilitate electric field distribution, the metal Zn tends to deposit inside the layer, easily resulting in a non-uniform plating behavior.^128^ In addition to carbon fibers, the electrospun polymer fibers also play an essential role in Zn anode protection. Compared with carbon, the polymer nanofiber protective layer can be formed *in situ* by the electrospinning method which avoids the utilization of the binder.^137^ More importantly, the thickness of the polymer fiber layer can be controlled by modulating the electrospinning time. Moreover, the polymer layer has a high flexibility and porous structure, and most of the polymer layer is ionically conductive but electronically insulating, which is beneficial for transporting Zn^2+^ across the interface layer and the uniform deposition of Zn^2+^.^138,139^ In fact, the polymer possesses numerous polar groups that serve as adsorption sites for Zn^2+^ transfer along the polymer chain to the reaction interface.^140^ Additionally, these groups facilitate the homogeneous distribution of Zn^2+^ at the molecular scale by enabling fast ion transport rates. Liu *et al.* reported an artificial interface (TPZA) with high ionic conductivity (19.8 mS cm^−1^) by permeating Zn-alginate (ZA) into porous thermoplastic polyurethane (TPU) fibers (Fig. 10a).^141^ As shown in Fig. 10b and c, owing to the protection of TPZA, the anode sustained the pristine morphology without the formation of by-products. For comparison, after 30 days, the Zn anode which was immersed in the electrolyte was randomly covered by the oriented hexagonal Zn_4_SO_4_(OH)_6_·3H_2_O. In addition to the property of anti-corrosion, the Zn^2+^ can transfer along the polymer chains of Zn-Alg, improving the transfer kinetics of Zn^2+^. Therefore, the Zn@TPZA//Zn@TPZA can be stably cycled over 1200 h at a current density of 5 mA cm^−2^ with a capacity of 5 mA h cm^−2^ (Fig. 10d).

A polybenzimidazole (PBI) nanofiber with abundant N-containing functional groups can promote the uniform deposition of Zn. Jian *et al.* constructed a PBI framework on the surface of Cu foil by an electrospinning method to serve as the substrate for Zn deposition, promoting uniform nucleation of Zn and achieving a dendrite-free Zn anode.^47^ The PBI nanofiber host with polar amine groups and porous structure can promote the permeation of electrolytes in the electrode. As illustrated in Fig. 10e, during the plating of Zn, the amine groups can act as nucleation seeds to guide the Zn to evenly deposit on the pores of the PBI nanofiber substrate to inhibit the formation of Zn dendrites. Consequently, at a current density of 10 mA cm^−2^, the Zn@PBI-Cu anode showed a compact surface without the vertical growth of zinc dendrites after 100 cycles (Fig. 10f). Besides, at a current density of 1 A g^−1^, the Zn@PBI-Cu//MnO_2_ displayed high capacity retention (close to 100%) and a high coulombic efficiency of about 100% after 100 cycles (Fig. 10g). Although this polymer fiber shows outstanding ability to suppress the growth of Zn dendrites, these nonconductive layers exhibit a huge impedance of interfaces which is not conducive to the rate capability of AZIBs.^142^ The reported electrospun fibers in the anode and their corresponding electrochemical performance are summarized in Table 4.

**Table 4:** A summary of electrospinning nanofibers for the Zn anode of AZIBs

Anode	Role of fibers	Electrospinning solution (precursor/polymer/solvent)	Structure	Voltage hysteresis	Life span	Coulombic efficiency	Cycling stability	Ref.
Zn@CNF	Substrate	PAN/DMF	Porous	Less than 20 mV	488 h at 0.5 mA cm^−2^	98.3% at 0.5 mA cm^−2^ (126 cycles)	92.1% at 0.5 A g^−1^ after 260 cycles	45
ZnCNF		PAN/DMF	—	23.9 mV	400 h at 0.1 mA cm^−2^	—	89.4% at 1C after 300 cycles	124
3DN-C@Zn		CO(NH_2_)_2_, Si(OC_2_H_5_)_4_/PAN, PMMA/DMF	—	34 mV	1000 h at 1 mA cm^−2^	99% after 300 cycles	∼100% at 1 A g^−1^ after 200 cycles	130
Sn-PCF@Zn		SiO_2_/PAN/DMF	Hollow	47 mV	500 h at 10 mA cm^−2^	99.8% at 10 mA cm^−2^ after 1000 cycles	73.5% at 10 A g^−1^ after 2500 cycles	134
Zn@CoCC		—	Hierarchical	—	800 h at 20 mA cm^−2^	—	—	132
Zn@Ni@AgNFs		PVA/H_2_O	Core/shell	—	—	—	90% at 0.13 mA cm^−2^ after 500 cycles	143
ZnO_*x*_@PCNF/Zn		ZIF-8/PAN/DMF	—	34 mV	250 h at 1 mA cm^−2^	99.3% at 1 mA cm^−2^ after 150 cycles	75% at 1C after 100 cycles	136
Zn@HSTF		C_16_H_36_O_4_Ti, C_8_H_20_O_4_Si/PVP/ethanol, acetic acid	Hollow	154 mV	200 h at 20 mA cm^−2^	99.54% at 20 mA cm^−2^ after 100 cycles	85% at 1 A g^−1^ after 1000 cycles	35
Sn@NHCF-Zn		SSR/PAN/DMF	Hollow	21 mV	370 h at 1 mA cm^−2^	99.7% at 5 mA cm^−2^ after 100 cycles	—	58
Cu/Zn-N/P-CMFs-Zn		PA-ZnCu NBs/PAN/DMF	Hollow	44.9 mV	630 h at 2 mA cm^−2^	98.2% at 10 mA cm^−2^ (900 cycles)	88.8% at 1 A g^−1^ after 2500 cycles	42
Cu NBs@NCFs-Zn		CuS NBs/PAN/DMF	Hollow	34.6 mV	450 h at 2 mA cm^−2^	98.8% at 5 mA cm^−2^ (1000 cycles)	67.6% at 1 A g^−1^ after 2000 cycles	46
Zn@PBI-Cu		PBI/DMAc	—	35 mV	Over 300 h at 10 mA cm^−2^	—	∼100% at 1 A g^−1^ after 1000 cycles	47
Zn@PAN-Cu		PAN/DMF	—	—	∼270 h at 2 mA cm^−2^	∼100% at 2 mA cm^−2^	—	144
Zn@PNF-Cu		P(VDF-TrFE), pyridine formate buffer/DMF	—	—	250 h at 10 mA cm^−2^	99.2% at 1 mA cm^−2^ (after 300 cycles)	—	145
Zn anode with a CNF interlayer	Protective layer	PAN/DMF	—	—	1200 h at 5 mA cm^−2^	99% at 1 mA cm^−2^ (60 cycles)	108.3% at 1 A g^−1^ after 420 cycles	33
Cu@CNFs-Zn		Cu(CH_3_COO)_2_·H_2_O/PAN/DMF	—	58 mV	2200 h at 1 mA cm^−2^	99.9% at 5 mA cm^−2^ (600 cycles)	—	135
Zn@MCFs		ZIF-8/PMMA, PAN/DMF	Hollow capsule-like	—	2500 h at 1 mA cm^−2^	99.67% at 2 mA cm^−2^ (1000 cycles)	82.8% at 1 A g^−1^ after 600 cycles	40
Zn@TPZA		TPU/DMF	—	—	1200 h at 5 mA cm^−2^	99.05% at 5 mA cm^−2^ (300 cycles)	—	141
β-PVDF-Zn		PVDF/DMF, acetone	Porous	40 mV	850 h at 0.5 mA cm^−2^	—	—	146

### 4.3 Separators

High-performance AZIBs depend on the synergy of all components. The separator acts as a carrier for the electrolyte, controlling the transport of ions, which determines the performance of the battery. Glass fiber separators are widely applied in AZIBs due to their high wettability, high ionic conductivity (about 17.3 mS cm^−1^ after absorbing electrolyte), and abundant porous structure. However, the metal Zn deposit in these pores of the glass fiber separator cannot be entirely converted to Zn^2+^ in the stripping process, ultimately resulting in the formation of “dead Zn”.^27^ Moreover, the glass fiber separator that absorbs excess electrolytes increases the total mass of the battery resulting in a low energy density.^26^ Although filter paper and non-woven fabric separators possess excellent mechanical properties and high porosity, their further application is prevented by the poor transport regulation ability.^147^ An ideal separator for AZIBs should not only have excellent ionic conductivity after taking in the electrolyte but should also regulate the transport of Zn^2+^ during the cycling process and prevent the growth of Zn dendrites. Compared to conventional separators, electrospun polymer fiber separators have attracted extensive attention because of their thermal stability, mechanical merit, electronic insulation, high mechanical flexibility, and controllable structure.^148^ In addition, the functional groups in the polymer fiber can promote the formation of coordination bonds with Zn^2+^, homogenizing the deposition of Zn^2+^ and suppressing the formation of Zn dendrites.^41^

#### 4.3.1 Pure polymer separators

**Fig. 11:**
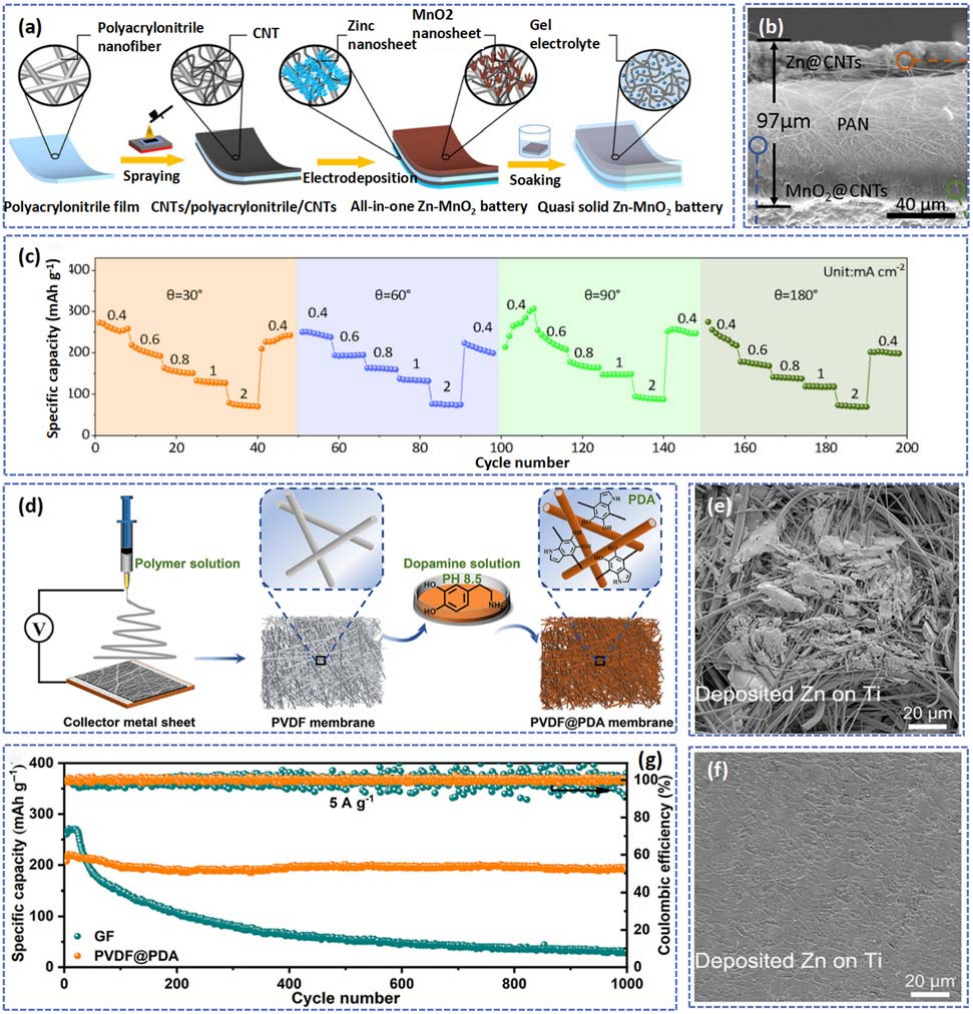
(a) Diagrammatic sketch showing the fabrication procedure of the AZIB. (b) SEM image of the cross-sectional view of the individual AZIB. (c) Rate performance of the AZIB cell at variational bending angles of 30, 60, 90, and 180°. Adapted from ref. 151, copyright 2021, American Chemical Society. (d) Schematic illustration of the fabrication process of the PVDF@PDA separator. SEM images of the Ti foils after Zn deposition at 2 mA cm^−2^ and 5 mA h cm^−2^ in Zn/Ti asymmetric cells with (e) a GF separator and (f) PVDF@PDA separator. (g) Long cycling performance of batteries with different separators at 5 A g^−1^. Adapted from ref. 41, copyright 2022, The Authors.

Owing to its excellent electrochemical stability, PAN has often been used to fabricate electrospun fiber separators.^149,150^ To stabilize the Zn anode, Liang's team synthesized a 3D long-range ordered PAN separator.^34^ Compared to the glass fiber separator (640.8%), the lower electrolyte uptake value (430.3%) of PAN film is advantageous for improving the energy density of the battery. Furthermore, the abundant –CN functional groups in the fibers not only promoted the electric field uniform distribution but also combined with Zn^2+^ to guide the uniform deposition of Zn^2+^ and effectively inhibit the growth of Zn dendrites. Benefiting from the mechanical flexibility, the PAN film was used as the separator and the current collector to prepare novel “paper-like” AZIBs with an all-in-one structure.^151^ As displayed in Fig. 11a and b, the Zn and MnO_2_ nanosheets were closely deposited on both sides of PAN which was modified by carbon nanotubes to form a cell with a thickness of about 97 μm, accelerating the transfer of electrons and achieving rapid kinetics. Therefore, the full cell exhibited a high capacity retention of about 98.7% after 500 cycles at 1 mA cm^−2^. In addition, at a bending angle of 180°, the battery also showed a high discharge capacity after being cycled at various current densities, indicating an excellent rate performance and outstanding flexibility (Fig. 11c).

#### 4.3.2 Hybrid polymer separators

Although a pure polymer film with high porosity and large SSA can be prepared by the electrospinning method, the poor mechanical strength has limited its application in flexible devices. Compared with pure polymer separators, hybrid polymer separators are prepared by mixing different types of substances by the electrospinning method (or pure polymer separators are modified by functional materials) which can promote the uniform deposition of Zn^2+^ and improve the mechanical strength of separators due to the multi-functional role and synergistic effect of the newly formed hybrids. For example, Saisangtham *et al.* used highly flexible polyurethane (PU) as the raw material to prepare PAN/bio-based PU separators by the electrospinning method.^152^ Besides, they investigated the effects of electrospinning solution concentration and parameters on the separators. The results revealed that the PAN separator modified by PU had a tensile strength of 44.16 MPa, which is much higher than that of the pure PAN membrane.

Moreover, some functional materials including graphene oxide (GO),^48^ sulfonated polysulfone (SPSF),^155^ and MXene^154^ have been added to regulate the flux of Zn^2+^. Among them is the strong interaction between the functional groups in polydopamine (PDA) and Zn^2+^, which promotes the transport of Zn^2+^ on the surface between the separator and electrolyte. Zhou's group developed a PDA functionalized PVDF (PVDF@PDA) to uniformize the homogeneous distribution of Zn^2+^ and suppress the formation of Zn dendrites (Fig. 11d).^41^ These abundant polar functional groups (–OH and –NH–) in the PDA improved the hydrophilicity of PVDF@PDA as well as favoring the formation of Zn–O and Zn–N coordination bonds with Zn^2+^. According to density functional theory calculations, the Zn–O and Zn–N can function as nucleation seeds to decrease the nucleation barrier of Zn and guide the ordered deposition of Zn^2+^. Herein, compared with the glass fiber separator (Fig. 11e), the surface of Ti foil with the functional separator was even without agglomeration and cracks at 2 mA cm^−2^ and 5 mA h cm^−2^ (Fig. 11f). Besides, the PVDF@PDA hybrid separator can effectively prevent the shuttling of V-species by formation of the V–O coordination bond during cycling. Therefore, as demonstrated in Fig. 11g, the Zn//NH_4_V_4_O_10_ full cell with the PVDF@PDA separator exhibited a high capacity retention of 92.3% after 1000 cycles at 5 A g^−1^.

Poly(*m*-phenylenedicarboxamide) (PMIA) with abundant amide groups, electrolyte affinity, and outstanding mechanical strength has been used as the separator for Li metal batteries.^156^ Inspired by this, Hu *et al.* fabricated a hybrid SPSF@PMIA (SP) nanofiber separator to stabilize the Zn anode.^155^ The abundant hydrophilic –SO_3_^−^ in SPSF and the N atom in PMIA with electronegativity will repel anions, which limit the migration of anions and enable the fast transfer of Zn^2+^. Therefore, compared with the batteries with PMIA (glass fiber or SPSF), the Zn/SP/Zn showed a higher Zn^2+^ transfer number (*t*_Zn_^2+^) of 0.74, which benefits the fast ion diffusion and fast charge transfer processes. Besides, owing to the strong ability of –CO–NH– in PMIA to absorb Zn^2+^ and the zincophilicity of –SO_3_^−^ in SPSF, the battery with the SP separator demonstrated a stable cycling ability and rate performance. Table 5 summarizes the polymer nanofiber separator performance.

**Table 5:** A summary of electrospinning nanofibers for separators of AZIBs

Separator	Electrospinning solution (precursor/polymer/solvent)	Electrolyte uptake	Ionic conductivity	Tensile strength	Life span	Cycling stability	Ref.
PAN	PAN/DMF	430.3%	0.45 × 10^−2^ S cm^−1^	—	800 h at 0.283 mA cm^−2^	84.3% at 5 A g^−1^ after 1000 cycles	34
PAN	PAN/DMF	—	—	—	—	98.7% at 1 mA cm^−2^ after 500 cycles	151
PVA-PAA	PVA, PAA/H_2_O	—	—	—	—	80% at 1 A g^−1^ after 2000 cycles	153
PAN/bio-based PU	PAN, bio-based PU/DMF	1971%	3.11 mS cm^−1^	44.16 MPa	250 h at 1 mA cm^−2^	—	152
PVDF@PDA	PVDF/DMF and DMAC (1 : 1 by vol)	403%	13.9 mS cm^−1^	9.7 MPa	Over 200 h at 2 mA cm^−2^	92.3% at 5 A g^−1^ after 1000 cycles	41
PG	GO/PAN/DMF	2267%	7.69 mS cm^−1^	7.1 MPa	13 000 h at 1 mA cm^−2^	71.5% at 2 A g^−1^ after 2800 cycles	48
PAN/bio-based PU/Ti_3_C_2_T_*x*_ MXene	Ti_3_C_2_T_*x*_ MXene/PAN, bio-based PU/DMF	2214 ± 49%	3.35 mS cm^−1^	1.68 MPa	—	—	154
SP	SPSF, PMIA/DMF, DMAc	810%	19.9 mS cm^−1^	2.9 MPa	Over 1000 h at 1 mA cm^−2^	80.8% at 0.5 A g^−1^ after 1000 cycles	155

## 5 Summary and perspectives

In conclusion, the reasons for the outstanding properties of the electrospun nanofibers are as follows. First, electrospun carbon fibers with large SSA and high conductivity can improve the electronic conductivity of materials and promote the diffusion of electrolyte in electrodes, which improve the rate performance and cycling ability of the battery. Second, these materials play a momentous role in maintaining the structural stability of electrodes. The porous (or hollow) structure can accommodate the Zn deposition and prevent the volume variation of the anode. In addition, the dissolution of active materials can be suppressed by forming a physical protective layer. Third, electrospun fibers with high porosity and flexibility can be used as binder-free and bendable electrodes, promising for bendable and wearable devices.

In this review, we summarized the recent progress of electrospinning nanofibers in AZIBs, focusing on vanadium-based materials, manganese-based materials, other cathode materials, carbon fiber-based and polymer fiber substrates, Zn anode protective layer, and polymer separators. In addition, we briefly introduced the principle and processing of the electrospinning technique and structural design of the electrospun fibers. Despite electrospinning fibers having made some research progress in AZIBs, several challenges still remain to be addressed. Therefore, to broaden the application of electrospun nanofibers, the following suggestions should be considered.

### 5.1 Precise preparation of functional fibers

The microstructure and properties of the electrospun fibers are related to the precursor solution, electrospinning parameters, and subsequent electrospinning process. However, very few studies have investigated the relationship between various parameters and the performance of fibers in AZIBs. Besides, various zincophilic units (such as functional groups, metal nanoparticles, metal oxides, and heteroatoms) have been reported to improve the zincophilicity and hydrophilicity of fibers to facilitate the homogeneous deposition of Zn. Sometimes, excessive zincophilic materials tend to accumulate together, which not only does not homogenize the Zn deposition but also changes the Zn deposition behavior, resulting in a severe growth of Zn dendrites. Thus, the preparation parameters of electrospun fibers should be systematically investigated and optimized.

### 5.2 In-depth investigation of the mechanisms

The working mechanism of the fiber material cannot be explained simply as the uniform distribution of the electric field on the surface of the Zn anode, the regulation of the flux of zinc ions, and the zincophilicity of the modified material. Specific experimental evidence should be provided. Moreover, some advanced characterization techniques including *in situ* optical microscopy (OM), *in situ* electron microscopy (EM) and *in situ* neutron depth profiling (NDP) and imaging can be used to elucidate the Zn growth mechanism. Analysis of the Zn metal is crucial to understanding the failure mechanism of AZIBs. Considering that the dynamics of electrochemical processes are difficult to observe during cycling, theoretical calculation can be used to further understand the mass transfer process of Zn^2+^.

### 5.3 Establishing the test standards

Although electrospun nanofiber electrodes show an impressive long cycle life at a small current density, it is difficult to meet the requirements of commercial applications. Moreover, different standards were used to test the batteries in previous studies, making it difficult to objectively evaluate various modification strategies. Therefore, it is important to establish unified test standards, which will facilitate the application of AZIBs. Besides, the electrochemical performance of the battery over a wide range of temperatures should be provided to promote the practical application of AZIBs in all climates.

### 5.4 Promoting large-scale commercial application of electrospinning technology

Electrospinning technology provides new insights into improving the performance of batteries. However, it is difficult to apply in industrial production on a large scale due to the use of toxic and corrosive solvents, expensive precursors, and lower production efficiency. Therefore, improving production efficiency, developing low-toxicity and environmentally friendly solvents, and exploring new types of and inexpensive polymer precursors are the main development directions for the future.

## Author contributions

All authors contributed to the writing of the manuscript and approved the final version of the manuscript.

## Conflicts of interest

The authors declare no conflict of interest.

## Supplementary Material
